# Upper Limb Evaluation in Duchenne Muscular Dystrophy: Fat-Water Quantification by MRI, Muscle Force and Function Define Endpoints for Clinical Trials

**DOI:** 10.1371/journal.pone.0162542

**Published:** 2016-09-20

**Authors:** Valeria Ricotti, Matthew R. B. Evans, Christopher D. J. Sinclair, Jordan W. Butler, Deborah A. Ridout, Jean-Yves Hogrel, Ahmed Emira, Jasper M. Morrow, Mary M. Reilly, Michael G. Hanna, Robert L. Janiczek, Paul M. Matthews, Tarek A. Yousry, Francesco Muntoni, John S. Thornton

**Affiliations:** 1 Dubowitz Neuromuscular Centre, UCL Institute of Child Health and Great Ormond Street Hospital, London, United Kingdom; 2 MRC Centre for Neuromuscular Diseases, UCL Institute of Neurology, London, United Kingdom; 3 Neuroradiological Academic Unit, UCL Institute of Neurology, London, United Kingdom; 4 Population, Policy and Practice Programme, UCL Institute of Child Health and Great Ormond Street Hospital, London, United Kingdom; 5 Institut de Myologie, GH Pitié-Salpêtrière, Paris, France; 6 GlaxoSmithKline, London, United Kingdom; 7 Division of Brain Sciences and Centre for Neurotechnology, Imperial College London, United Kingdom; University of Rome La Sapienza, ITALY

## Abstract

**Objective:**

A number of promising experimental therapies for Duchenne muscular dystrophy (DMD) are emerging. Clinical trials currently rely on invasive biopsies or motivation-dependent functional tests to assess outcome. Quantitative muscle magnetic resonance imaging (MRI) could offer a valuable alternative and permit inclusion of non-ambulant DMD subjects. The aims of our study were to explore the responsiveness of upper-limb MRI muscle-fat measurement as a non-invasive objective endpoint for clinical trials in non-ambulant DMD, and to investigate the relationship of these MRI measures to those of muscle force and function.

**Methods:**

15 non-ambulant DMD boys (mean age 13.3 y) and 10 age-gender matched healthy controls (mean age 14.6 y) were recruited. 3-Tesla MRI fat-water quantification was used to measure forearm muscle fat transformation in non-ambulant DMD boys compared with healthy controls. DMD boys were assessed at 4 time-points over 12 months, using 3-point Dixon MRI to measure muscle fat-fraction (f.f.). Images from ten forearm muscles were segmented and mean f.f. and cross-sectional area recorded. DMD subjects also underwent comprehensive upper limb function and force evaluation.

**Results:**

Overall mean baseline forearm f.f. was higher in DMD than in healthy controls (p<0.001). A progressive f.f. increase was observed in DMD over 12 months, reaching significance from 6 months (p<0.001, n = 7), accompanied by a significant loss in pinch strength at 6 months (p<0.001, n = 9) and a loss of upper limb function and grip force observed over 12 months (p<0.001, n = 8).

**Conclusions:**

These results support the use of MRI muscle f.f. as a biomarker to monitor disease progression in the upper limb in non-ambulant DMD, with sensitivity adequate to detect group-level change over time intervals practical for use in clinical trials. Clinical validity is supported by the association of the progressive fat transformation of muscle with loss of muscle force and function.

## Introduction

Duchenne Muscular Dystrophy (DMD) is an X-linked recessive disorder affecting 1 in 5000 male births.[1] Mutations in the *DMD* gene result in the absence of the sarcolemmal protein dystrophin, which leads to progressive muscle damage and fat replacement. Clinically, DMD patients become wheel chair bound by their mid-teens and die prematurely of cardiac/respiratory failure.[2, 3]

Glucocorticoid therapy is currently the only pharmacological intervention proven to delay disease progression in muscles,[4, 5] however a number of promising novel experimental therapies are at different clinical trials stages, e.g. antisense oligomer (AO) mediated exon skipping, which aims to restore semi-functional protein products; and drugs to induce read-through nonsense mutations.[6–8] Primary outcome measure in clinical trials currently rely on invasive endpoints such as muscle biopsy to describe restoration of dystrophin in muscle fibres, providing only limited sampling of the pathology, or functional tests with restricted applicability and variable reproducibility. For instance, the 6-minute-walk test (6-MWT) has been utilized as a primary outcome end-point in phase II-III clinical trials;[8–10] however limitations of this functional test include that it is motivation dependent[11] and can only be used for ambulant boys. In addition, invasive endpoints such as muscle biopsy to assess restoration of dystrophin in muscle fibres is often used as a pharmacodynamics marker, providing however only limited information due to sampling limitation. Thus non-invasive and objective trial outcome measures applicable in ambulant and non-ambulant subjects are urgently required. Recent studies have shown that magnetic resonance imaging (MRI) and spectroscopy (MRS) provide sensitive markers of muscle pathology and disease progression in the lower limbs of DMD subjects, [12–14], suggesting MRI-quantified muscle fat content as a promising candidate biomarker. However, to date limited information is available regarding the potential of these methods to assess upper limb disease progression, although initial data are encouraging: Wary et al. recently reported forearm MRI measurements in non-ambulant boys with DMD, observing a significant correlation between forearm fat infiltration and time non-ambulant.[15]

The aim of our longitudinal study was to examine the ability of MRI muscle-fat measurement to quantify disease progression in the upper limbs of non-ambulant DMD boys, and to compare the changes over 3, 6 and 12 months of MRI fat measurement compared with muscle strength and validated functional assessments.

## Methods

### Participants

We recruited 15 non-ambulant DMD boys with a mean age of 13.3 y, (range: 10.8–17.3y.) mean duration of non-ambulation 20.2 months (range: 4.7–41.6 months) and mean BMI 26.5 (range: 20.8–41.7). Assessments were performed at baseline, 3, 6 and 12 months; 6 subjects withdrew from the study due to the frequent hospital visits, which were challenging to sustain in the absence of therapeutic intervention.

At the time of the study all but one subject were receiving glucocorticoids. Ten age and gender-matched healthy control subjects were scanned once (mean age: 14.6y, range: 13–17y; mean BMI 21.5, range: 16.5–25.4). To assess measurement reproducibility 4 healthy controls were re-tested at 6 weeks.

Written informed consent was obtained from the parents or guardians on behalf of the minors/children enrolled in this study. The Brighton & Sussex Research National Ethics Committee approved the consent procedure and this study, which was performed in compliance with the Declaration of Helsinki.

### MRI acquisition and data analysis

Unilateral upper-limb MRI was performed at 3T (Siemens Skyra; Siemens, Erlangen, Germany) using a flexible surface matrix coil (4-Channel Flex Coil) wrapped around the dominant forearm. Subjects lay in the scanner in the head-first supine position, with the dominant arm to be imaged lying in a comfortable position on the scanner bed alongside the torso. Three-point-Dixon[16] images were acquired (2D gradient-echo TE1/TE2/TE3/TR = 3.45/4.60/5.75/102ms, flip angle 10°, nine 6mm axial slices, slice gap 12mm, FOV 18x18cm, matrix 320×320, pixel size 0.56×0.56mm, NEX = 4).

Images were post-processed offline with a Python programming language pipeline according to Glover and Schneider’s algorithm[17] assuming a single-peak fat component, and separated fat (f) and water (w) images were used to calculate pixel-wise fat fraction (f.f.) maps according to $f.f.\;=\;\frac{f}{w+f}\times 100\%$.

**Fig 1:**
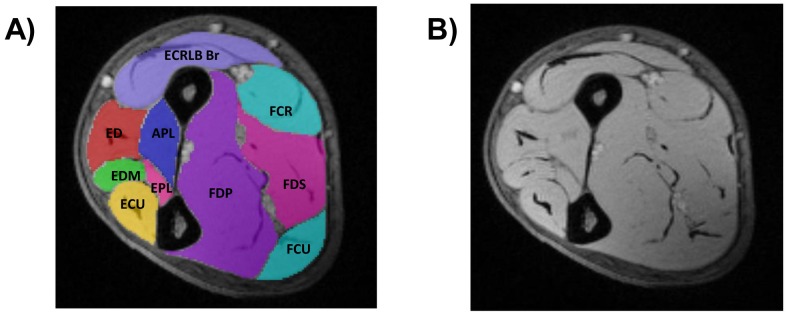
Muscle segmentation (A) and raw 3-point Dixon (B) of the central slice in the dominant forearm of a healthy control. **DORSAL compartment**: Extensor carpi ulnaris (ECU), extensor digiti minimi (EDM), extensor digitorum (ED), extensor pollicis longus (EPL), abductor pollicis longus (APL), extensor carpi radialis longus/brevis and brachioradialis (ECRLB Br). **VOLAR compartment**: flexor digitorum profundus and flexor pollicis longus (FDP), flexor digitorum superficialis and palmaris longus (FDS), flexor carpi ulnaris (FCU), flexor carpi radialis (FCR).

Observers blinded to diagnosis manually defined individual muscle-group regions of interest (ROI) on the TE = 3.45ms Dixon image to encompass each muscle-group boundary to the fascia using the ITK Snap software.[18] For consistency all ROI definitions were subsequently reviewed by a single blinded observer (MRBE). For each subject 3 of the 9 available baseline image slices were chosen for analysis: a central slice defined as the first axial slice distal to the supinator muscle, and proximal and distant slices centred ±74mm relative to the central slice. For consecutive longitudinal data from the same subject, the slices selected for analysis were carefully matched to the baseline slices by visual inspection and reference to a coronal scout image. On each slice 10 forearm muscle-group ROIs were defined (Fig 1).

Muscle ROIs on follow-up scans were defined in each case with reference to the respective baseline muscle segmentation for that subject to maintain longitudinal anatomical consistency. The segmented ROI were transferred to the intrinsically co-registered f.f. maps, and visually inspected for placement errors or the presence of gross artefact, and adjusted if necessary. For all individual ROIs, custom-written software extracted individual muscle-group ROI mean f.f. and muscle compartment cross-sectional area (CSA) in mm^2^. For each subject, at each of the distal, central and proximal slice levels the following summary measures were determined: *total muscle compartment f*.*f*., calculated as the weighted-mean of the individual muscle-group mean f.f.s, each ROI f.f. weighted by its respective CSA; *total muscle compartment cross-sectional area* calculated as the sum of the individual muscle CSAs, and *remaining muscle area* calculated as:

$$cross-sectional\;muscle\;compartment\;area\;\times \;\frac{100-total\;muscle\;compartment\;f.f.}{100}.$$

### Functional assessment

In a clinical trial compatible mode, each subject was assessed as follows: 1) performance of upper limb (PUL) module, a validated 74 point functional scale for motor performance relating to everyday life activity;[19, 20]; 2) muscle strength for shoulder flexion, elbow extension and flexion, and wrist extension measured in lbs using a hand-held myometer (Microfet, Hoggan, UT); 3) MyoSet (Myopinch, Myogrip and Moviplate), a suite of recently validated novel tools to assess strength and fatigability of the upper limb;[21, 22]; 4) Egen Klassification (EK2) interview version 2, which is used to determine performance of tasks in daily life (total score = 51)[23]; 5) spirometry and peak cough flow using a Vitalograph Pneumotrac 6800; and 6) record of time to loss of ambulation (LOA) in months.

### Statistical methods

Baseline MRI measures were compared between the DMD and healthy control groups using unpaired t-tests. In the DMD group, changes in clinical measurements and MRI scores were compared within boys over the 3 follow-up time points using mixed model regression analysis. This method is suitable for unbalanced data, obtained as a result of loss to follow-up and incomplete dataset, therefore accounting for different number of subjects at different time points and differing between MRI and physiotherapy assessment. Furthermore, a separate model was fitted for each outcome measurement with adjustment for length of time non-ambulant; results are presented as adjusted mean changes for the 3 month, 6 month and 12 month periods, with 95% confidence intervals (CI). The data for each measure were assessed for normality using the Shapiro-Francia W dash test and, where there was some evidence of non-normality, we investigated the use of appropriate transformations. Findings were found to be very similar for the transformed data and we therefore present results for untransformed data only. To account for multiplicity a P-value <0.01 was considered significant.

## Results

From the 15 subjects initially recruited, combined MRI and clinical assessment data were obtained for 9/15 subjects (mean age 13.2y; range: 11.1–17.6) at the 3 month follow-up and 7/15 at both 6 the month (mean age 13.9y; range: 11.6–17.9) and 12-month (mean age 14.2y; range: 11.8–18.2) time points. Although MRI was overall well tolerated, 6 subjects found the commitment to the study too demanding and withdrew before completing the full 12-month assessments. In addition, MRI from 4 patients at 6 and 2 patients at 12 months were excluded from the analysis due to subject-motion related image artefacts. Finally, MyoSet data could not be collected for 4 subjects at 3 months, 2 subjects at 6 months and 1 subject at 12 months because the tools were sent back to the manufacturer for yearly calibration or used in another protocol. One patient contributed to baseline evaluation only.

### Muscle MRI- fat transformation, cross-sectional area, and remaining non-fat area

The central slice overall mean total muscle compartment f.f. in the DMD subjects was significantly higher than for healthy controls (14.1%, 95% CI 8.4, 19.9 in DMD; 0.9%, 95% CI 0.7, 1.0 in healthy controls; p<0.001), as were individual muscle-group mean f.f. (Table 1 and Table A in S1 Table). The central slice mean total cross-sectional muscle compartment area was reduced in DMD (1702 mm^2^, 95% CI 1520, 1883 mm^2^) relative to the healthy controls (2389 mm^2^, 95% CI 1878, 2899 mm^2^; p = 0.006).

**Table 1:** Baseline characteristics of DMD subjects.

	Mean	95% CI
**CENTRAL SLICE TOTAL MUSCLE COMPARTMENT FAT FRACTION (%)**	14.1	8.4; 19.9
**PROXIMAL SLICE TOTAL MUSCLE COMPARTMENT FAT FRACTION (%)**	17.5	10.9; 24.1
**DISTAL SLICE TOTAL MUSCLE COMPARTMENT FAT FRACTION (%)**	19.7	12.5; 26.9
**CENTRAL SLICE DORSAL COMPARTMENT FAT FRACTION (%)**	14.1	8.7; 19.5
**CENTRAL SLICE VOLAR COMPARTMENT FAT FRACTION (%)**	14.2	7.1; 21.3
**ECRLB Br FAT FRACTION (%)**	31.2	20.5; 41.8
**CENTRAL SLICE TOTAL MUSCLE COMPARTMENT CROSS-SECTIONAL MUSCLE AREA (mm**^**2**^**)**	1702	1520; 1883
**CENTRAL SLICE TOTAL REMAINING (NON-FAT) MUSCLE AREA (mm**^**2**^**)**	1472	1254; 1690
**MYOPINCH (Kg)**	2.5	2.1; 2.9
**MYOGRIP (Kg)**	6.7	5.6; 7.8
**PERFORMANCE OF UPPER LIMB (Total score = 74)**	65.9	61.6; 70.3
**PERFORMANCE OF UPPER LIMB (Shoulder domain score = 16)**	9.9	6.8; 12.9
**MOVIPLATE (taps in 30 seconds)**	56.3	49.7; 62.9

Baseline means and 96% CI for the 15 DMD subjects

The extensor carpi radialis longus/brevis and the brachioradialis (ECRLB Br) group showed the greatest f.f. difference between DMD and the healthy controls (Table A in S1 Table), with an overall mean f.f. 31.2% (95% CI 20.5, 41.8%) in the DMD group vs healthy controls, which was 1.8% (95% CI 0.7, 2.8%).

**Fig 2:**
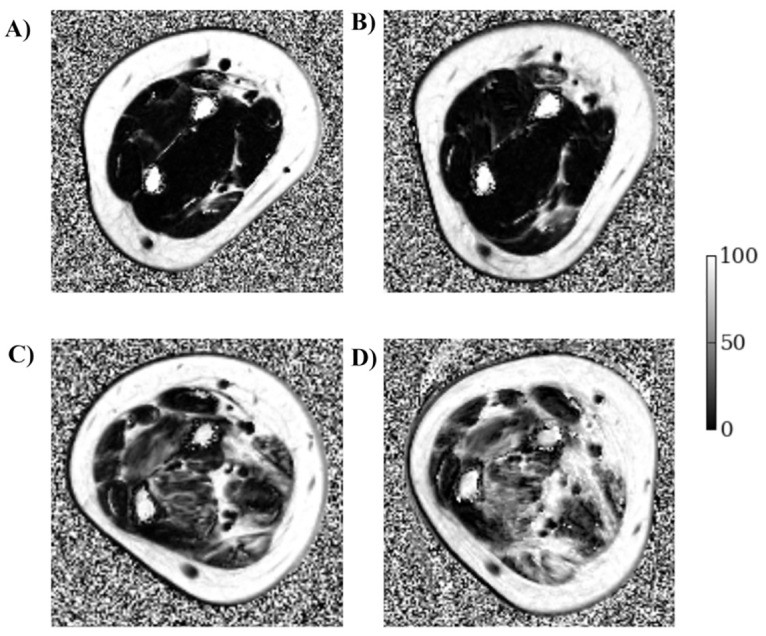
3-point Dixon fat-fraction (f.f.) maps of dominant forearm central slice at baseline (left images) and 12 months (right images). **Top:** 13 y.o. DMD, non-ambulant for 40 months, and on daily steroids. Overall mean f.f. at baseline = 7.6% (A) and 12 months = 9.7% (B). **Bottom:** 11 y.o. DMD, non-ambulant for 14 months, not on steroid therapy. Mean f.f. at baseline = 30.7% (C) and 12 months = 43.3% (D). (Grey-level bars represent f.f. from 0 to 100%).

**Fig 3:**
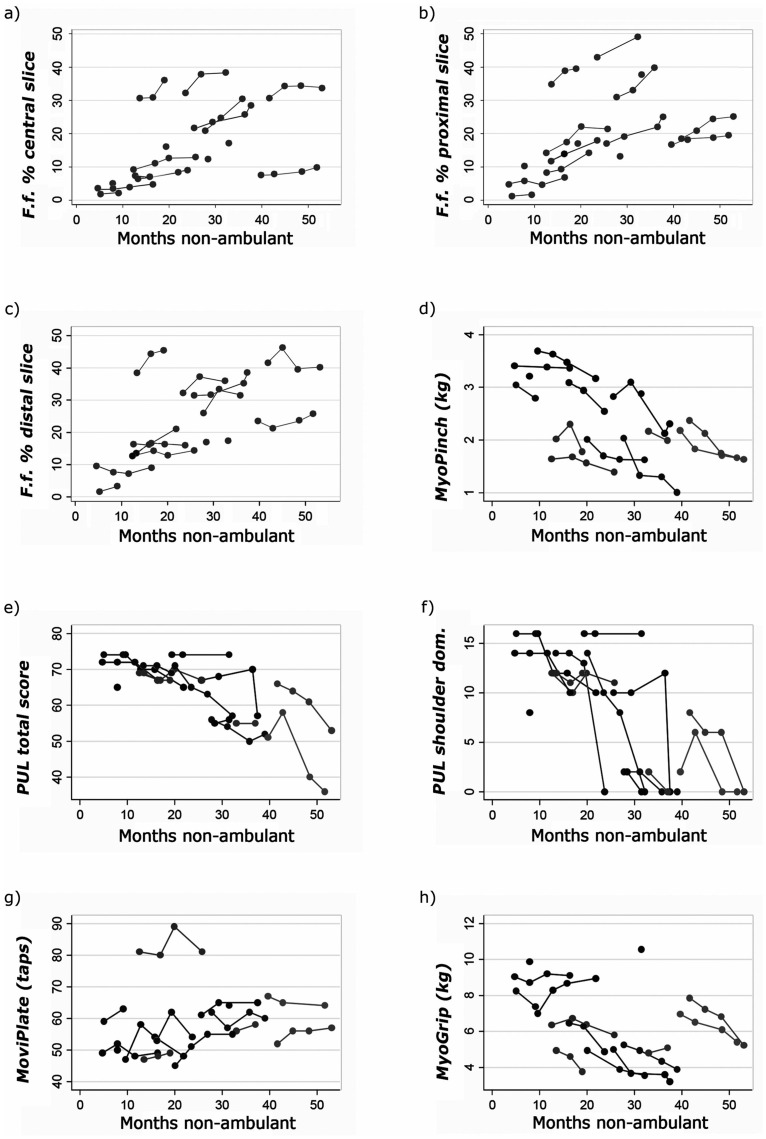
Plots of individual trajectories for (A) central slice overall muscle %fat fraction, (B) proximal slice overall muscle %fat fraction, (C) distal slice overall muscle %fat fraction, (D) MyoPinch, (E) total score for Performance of Upper limb, (F) PUL Shoulder domain score, (G) MoviPlate and (H) MyoGrip. f.f. = fat fraction. PUL = Performance of upper limb.

Longitudinally in the DMD group we observed a significant increase in central slice total muscle compartment f.f. of 3.9% units mean change (95% CI 1.9, 5.7%) above baseline at 6 months (p<0.001, n = 7). At 12 months, the mean increase was 5.0% units (95% CI 3.2, 6.9) above baseline (also n = 7) (Table 2, Figs 2 and 3a). Proximal and distal slice mean increases were also significant at 12 months: 6.9% (95%CI 5.2, 8.7%; p<0.001) and 4.3% (95%CI 2.0, 6.6%; p<0.001) respectively (Table 2, Fig 3b and 3c). At 12 months, the dorsal compartment was more affected than the volar compartment, with, at the central-slice level, a mean increase of 6.3% units (95% CI 3.6, 9.1; p<0.001) compared to the volar compartment (3.4%, 95% CI 0.9, 5.7; p<0.01) (Table 2). All individual muscle groups showed significant f.f. increase over one year (all p<0.001, Table B in S1 Table), with the ECRLB Br again being the most affected muscle group, with a mean f.f. increase of 7.0% (95% CI 4.1, 10.0; p<0.001) (Table 2).

**Table 2:** MRI and clinical indices mean changes (95% CI) from baseline from analysis of variance.

	3 months	6 months	12 months
**CENTRAL SLICE TOTAL MUSCLE COMPARTMENT FAT FRACTION (%)**			
**Mean change from baseline (95% CI)**	1.4	3.9	5.0
	(-0.3, 3.2)	(1.9, 5.7)	(3.2, 6.9)
**No. of subjects**	9	7	7
**P value**	0.11	< 0.001	< 0.001
**PROXIMAL SLICE TOTAL MUSCLE COMPARTMENT FAT FRACTION (%)**			
**Mean change from baseline (95% CI)**	2.1	4.5	6.9
	(0.5, 3.7)	(2.7, 6.3)	(5.2, 8.7)
**No. of subjects**	9	7	7
**P value**	0.01	< 0.001	< 0.001
**DISTAL SLICE TOTAL MUSCLE COMPARTMENT FAT FRACTION (%)**			
**Mean change from baseline (95% CI)**	2.2	2.2	4.3
	(0.06, 4.3)	(-0.05, 4.5)	(2.0, 6.6)
**No. of subjects**	9	7	7
**P value**	0.04	0.06	< 0.001
**CENTRAL SLICE DORSAL COMPARTMENT FAT FRACTION (%)**			
**Mean change from baseline (95% CI)**	1.7	5.5	6.3
	(-0.9, 4.4)	(2.7, 8.3)	(3.6, 9.1)
**No. of subjects**	9	7	8
**P value**	0.20	<0.001	<0.001
**CENTRAL SLICE VOLAR COMPARTMENT FAT FRACTION (%)**			
**Mean change from baseline (95% CI)**	1.3	0.7	3.4
	(-1.0, 3.7)	(-1.8, 3.3)	(0.9, 5.7)
**No. of subjects**	9	7	8
**P value**	0.27	0.58	<0.01
**ECRLB Br FAT FRACTION (%)**			
**Mean change from baseline (95% CI)**	1.7	6.1	7.0
	(-1.1, 4.5)	(3.1, 9.2)	(4.1, 10.0)
**No. of subjects**	9	7	8
**P value**	0.24	<0.001	<0.001
**CENTRAL SLICE TOTAL MUSCLE COMPARTMENT CROSS-SECTIONAL MUSCLE AREA (mm**^**2**^**)**			
**Mean change from baseline (95% CI)**	-5.4	42.1	140
	(-88.1, 77.4)	(-47.0, 131.2)	(50.9, 229.1)
**No. of subjects**	9	7	7
**P value**	0.90	0.36	< 0.01
**CENTRAL SLICE TOTAL REMAINING (NON-FAT) MUSCLE AREA (mm**^**2**^**)**			
**Mean change from baseline (95% CI)**	-28.4	-32.1	22.0
	(-93.6, 36.8)	(-102.6, 38.1)	(-48.2, 92.3)
**No. of subjects**	9	7	7
**P value**	0.39	0.37	0.54
**MYOPINCH (Kg)**			
**Mean change from baseline (95% CI)**	-0.1	-0.38	-0.50
	(-0.27, 0.03)	(-0.53, -0.22)	(-0.66, -0.34)
**No. of subjects**	10	9	8
**P value**	0.12	< 0.001	< 0.001
**MYOGRIP (Kg)**			
**Mean change from baseline (95% CI)**	-0.22	-0.50	-1.04
	(-0.70, 0.27)	(-1.01, 0.002)	(-1.56, -0.51)
**No. of subjects**	10	9	8
**P value**	0.38	0.05	< 0.001
**PERFORMANCE OF UPPER LIMB (Total score = 74)**			
**Mean change from baseline (95% CI)**	-0.5	-2.6	-9.2
	(-2.8, 1.9)	(-5.2, -0.03)	(-11.9, -6.4)
**No. of subjects**	14	11	9
**P value**	0.69	0.05	< 0.001
**PERFORMANCE OF UPPER LIMB (Shoulder domain score = 16)**			
**Mean change from baseline (95% CI)**	-0.7	-1.0	-6.9
	(-2.6, 1.1)	(-2.9, 0.9)	(-9.0, -4.9)
**No. of subjects**	14	11	9
**P value**	0.42	0.32	< 0.001
**MOVIPLATE (taps in 30 seconds)**			
**Mean change from baseline (95% CI)**	2.3	4.7	1.7
	(-0.02, 4.6)	(2.01, 7.4)	(-0.8, 4.3)
**No. of subjects**	11	9	8
**P value**	0.05	0.001	0.19

P value < 0.01 was considered significant. ECRLB Br = extensor carpi radialis longus/brevis and brachioradialis

Mean central slice total cross-sectional muscle area in the DMD subjects increased above baseline by 140 mm^2^ (95% CI 50.9, 229.1 mm^2^) at 12 months (p<0.01), although the total remaining (i.e. non-fat) muscle area, which at baseline was 1472 mm^2^ (95% CI 1254, 1690 mm^2^), did not show significant change (mean change 22 mm^2^, 95% CI -48, 92 mm^2^, p = 0.5).

Excluding the DMD boy who was not receiving steroids from the analysis did not change our statistical conclusions (S2 Table). In healthy controls, the central slice overall mean muscle f.f. change between baseline and 6 weeks was 0.2 (95%CI (-0.2, 0.5 p = 0.26, n = 4). Individual subject differences are reported in Table C in S1 Table.

### Clinical assessment

Functional and strength mean values at baseline are reported in Table 1.

In 9 DMD subjects, mean MyoPinch measured pinch strength decreased significantly from baseline by 0.38 kg (95% CI -0.53, -0.22; p<0.01, n = 9) at 6 months (Table 2, Fig 3e), and grip strength measured by MyoGrip had decreased significantly by 1.0 kg (95% CI -1.56, -0.51; p<0.001, n = 8) at 12 months from baseline. The PUL total score and shoulder domain index had both deteriorated significantly at 12 months, with group mean decreases of 9.2 (95% CI -11.9; -6.4; p<0.001) and –6.9 (95% CI -9.0; -4.9; p<0.001) respectively in 9 DMD subjects. Distal arm functional assessment using the MoviPlate detected a significant improvement of function at 6 months (4.69, 95% CI 2.0, 7.4; p = 0.001, n = 9) with subsequent decline (Table 2, Fig 3g). These conclusions remained unchanged on excluding the subject not treated with steroids (S2 Table). All other evaluations, including spirometry and muscle strength measurement with myometry, failed to reach statistical significance in our cohort. Detailed results are reported in the appendix (S3 Table and S1 Fig).

## Discussion

Our study suggests that upper limb forearm MRI fat quantification is a sensitive biomarker for disease progression in non-ambulant DMD boys. Not only could MRI discriminate DMD from healthy controls (p = 0.002), but longitudinally, group-wise increased forearm muscle-fat infiltration in the dominant forearm of DMD subjects was significant (p < 0.001) as early as 6 months from baseline. At one year, the DMD group mean central slice muscle f.f. increase was 5.0% (95% CI 3.2, 6.9%) above a baseline value of 14.1% (95% CI 8.4, 19.9%; p<0.001). In addition, we demonstrated that whilst in the patient group mean total muscle compartment cross-sectional area increased over 12 months, the remaining total non-fat muscle area within the muscle compartments did not change. We speculate that this apparent overall increase in volume of the muscle in part reflects the process of fat infiltration or tissue transformation in DMD, although it remains to be established to what extent muscle pseudo-hypertrophy in DMD is attributable to this cause. All muscle groups showed a significant f.f. increase over one year (all p<0.001), with the dorsal compartment more affected than the volar. The ECRLB Br was the most severely affected individual muscle group at baseline (mean f.f. 31.2, 95% CI 20.5; 41.8%) with the most marked f.f. increase over 12 months equal to 7.0% (95% CI 4.1, 10.0; p<0.001). The muscle fat transformation extended over the whole length of the lower arm: proximal and distal slices showed a similar progression of fat transformation, although the proximal slice was more severely affected, both across the total muscle cross-section, and within individual muscles.

Regarding the clinical evaluations, at 6 months we observed a significant decrease from baseline in MyoPinch-measured pinch strength of 0.38 kg (95% CI -0.53, -0.22, p<0.001). This was sustained at one year (-0.50 kg 95% CI –0.66, -0.34 kg from baseline, p<0.001). Over 12 months handgrip strength also significantly reduced by 1.0 kg (95% CI -1.56, -0.51 p<0.001). In line with previous publications, [21, 22] the MoviPlate, a tapping device designed to measure endurance of upper limb, showed a modest improvement over 6 months followed by a decline. This phenomenon has been observed in the early non-ambulant DMD phases (i.e. boys who are wheelchair-bound < 3years) [21] and may be explained by a distal upper limb functional training effect or by a motor strategy improvement following loss of ambulation with increased demands on arm function. The PUL scale showed highly significant loss of function at 12 months (p<0.001), with a mean drop in score of 9.2 (95% CI -11.9; -6.4) out of 74; this numerical result was predominantly driven by the loss of function in the shoulder domain. Interestingly, loss of shoulder-girdle muscle function in our study was associated with progressive lower arm muscle fat transformation, suggesting our observed changes reflect pathology not limited to the forearm muscles. Although specific muscle group related strength was measured, including shoulder extension, elbow flexion and extension, and wrist extension, these assessments did not yield any significant results within the study time frame in this relatively small patient cohort. None of the other assessments, including spirometry and a patients’ reported questionnaire changed significantly over 12 months.

When designing clinical trials for rare disorders, critical issues which need to be taken into account include: selecting an endpoint which is as observer and subject-motivation independent as possible; reducing the burden of clinical-trial participation, favouring non-invasive endpoints; and selecting an endpoint capable of identifying clinically meaningful changes in a small cohort of patients. MRI is being increasingly applied to neuromuscular disorders, not only for defining phenotype [24, 25], but also to provide biomarkers responsive to therapies. [26, 27] Studies have already demonstrated the suitability of MRI to characterise pathology in DMD, [12, 28] monitoring disease progression in the lower limbs [13, 14, 29] and in the upper limb of non-ambulant subjects. [15] Further studies have explored imaging in combination with lower extremity strength and/or functional assessment. [30–35] A recent study has shown that lower limb MRI and MRS can effectively detect the beneficial effect of glucocorticoid therapy both in cross-sectional comparison and at 3 months.[27] In relation to upper limb muscle MRI, to date, only one cross-sectional study reported on forearm fat transformation in 24 non-ambulant DMD subjects with deletions amenable to exon 53 skipping therapy, and showed a strong positive correlation between muscle fat content and disease severity expressed as time non-ambulant. [15]

Overall, MRI combines the advantages of being non-invasive and repeatable longitudinally, allowing investigators to have a deep understanding of the timing and the extent of muscle response to therapeutic intervention, in multiple muscle groups. This is in contrast to muscle biopsies, which are not only invasive procedure, but also limited to a small sample in a single muscle. There is also potential to combine multi-centre MRI data in multi-centre trials: international expert workshops under the auspices of the TREAT-NMD have proposed harmonized protocols for skeletal muscle MRI-based outcome measures in this context,[36] and a multicentre study evaluated the reproducibility among centres of MRI and MRS in lower limbs of DMD boys.[37] Of the available MRI readouts, a number of studies support the notion that the 3-point-Dixon technique [16, 38] may be the best currently available method to measure fat transformation in DMD associated with disease progression in DMD.[31, 36, 39] Indeed, lower-limb MRI and MRS have been included as exploratory endpoints in clinical trials for ambulant boys in patients treated with Drisapersen [24] and in the on-going EU FP7 funded SKIP-NMD programme, which aims to restore dystrophin with morpholino antisense oligomer exon skipping for patients with deletions skippable by exon 53, and which will yield results by 2016.[26, 40]

The current study, provides longitudinal MRI natural history data regarding fat transformation in the forearm of non-ambulant DMD, with corroborative detailed clinical evaluation: both MRI and Myopinch sensitively detected changes at 6 months and therefore both qualify as complementary outcome measures. MRI allows highly specific assessment of individual muscles within in a single examination while probing simultaneously across the different muscle groups at multiple levels, whereas Myopinch measures a specific function of strength reflecting the combined effort of one distinct muscle group.

Importantly, comparing our MRI measurements with the functional data supports the validity of MRI-determined forearm f.f. as a clinically-relevant marker of disease progression in DMD. The progressive fat transformation measured by MRI in our cohort may be associated not only with a decline in strength (for pinching at 6 months, and gripping at 12 months), but also in functional impairment as measured by the PUL at 1 year; we therefore conclude that f.f. increase is a clinically meaningful index of pathology.

The high responsiveness of f.f. to pathological change in DMD with the ability to detect significant change at as early as 6 months in a small cohort of patients, should allow adequately powered clinical trials with shorter duration, and or with reduced sample size requirements than those based upon conventional clinical end-points alone. For example, in our study we observed total cross-sectional muscle f.f. change at 6 months from baseline of approximately 4% (± 3%) in 7 DMD subjects. In a hypothetical treatment trial, if this change was compared with an expected mean change (treatment effect size) of 0.5% in a treatment arm,[27] and assuming a similar SD, a power of 90% and significance level of 5%, a minimum of 16 patients per group would be required. This would considerably reduce trial sample size and duration compared to studies using, for example, the 6-MWT as a clinical end-point. Furthermore, our data also supports the feasibility of extending eligibility for DMD treatment trial inclusion to non-ambulant as well as ambulant, since disease progression, and therefore we may assume treatment response, can be quantified by MRI in the upper limb.

Our study protocol was well tolerated overall, allowing adequate rest between the image acquisition and physiotherapy assessment. The 6 subjects who withdrew from the study reported that the frequent hospital visits (i.e. 4 in 12 months) were difficult to sustain in absence of therapeutic intervention. Cooperation in lying still is essential for MRI, and indeed 4 of our subjects moved during imaging necessitating exclusion of those data. Reducing the overall MRI examination time by excluding non-essential acquisition sequences or exploiting MRI acceleration strategies [41] may improve patient compliance and reduce the study burden in future studies. However, MRI results are nevertheless likely far less subject motivation-dependent than conventional primary endpoints such as the 6-MWT used in ambulant clinical trials, in which performance may be affected by the level of tiredness or motivation on the day.

Limitations of our study include the relatively small sample size, exacerbated by the number of subjects who withdrew from the study, with inclusion restricted to a selected patient population (i.e. non-ambulant and below 18 years of age). It is likely that the measures we investigated may well vary according to age at baseline and length of time non ambulant, and it would be of interest to adjust for these factors in a future larger study. Although it was not our objective to explore the effect of treatment including steroids on MRI response, this may be important; excluding data from the single patient not on steroid therapy from our analysis did not alter our findings. Finally, our MRI coverage was in this study limited to the dominant forearm, and the f.f. analysis to three slices; future studies will address pathology and disease progression in limb regions and muscle groups not included in this work.

In conclusion, our study provides novel data to inform rational imaging endpoint specification and trial design for studies in non-ambulant Duchenne cohorts. Although our cohort of boys was relatively small, we were able to document objective changes in MRI measures of muscle fat transformation and validate their clinical relevance by demonstrating a strong association with measures of muscle force, function and disease progression. The time course of the measures’ response to deterioration suggests detection of disease stabilization or improvement over periods as short as 6–12 months is readily achievable in small cohorts. The complementarity of the methods suggests a mechanistic relationship between muscle tissue fat transformation, muscle force production and overall function.

## Supporting Information

S1 Fig (A) Individual trajectories for central slice total cross sectional muscle area in mm2 and (B) total remaining muscle area in mm2, (C) wrist extension with microfet myometer (lb), (D) Egen Klassification (EK2), (E) Forced vital capacity (ltr) (F), Peak cough flow (ltr/sec). (DOCX)

S1 Table Individual muscle-group mean fat fraction (%) for the central slice in each DMD and healthy control (HC) subject at baseline (a) and mean (95%CI) changes from baseline in DMD (b). Central slice mean f.f. at baseline and 6 weeks in healthy controls (HC) (c).
(DOCX)

S2 Table MRI and clinical indices: mean (95%CI) changes from baseline; analysis of variance excluding the DMD subject not taking steroid therapy. (DOCX)

S3 Table Clinical indices mean changes (95% CI) from baseline from analysis of variance. (DOCX)
