# Supplementary material for: Upper Limb Evaluation in Duchenne Muscular Dystrophy: Fat-Water Quantification by MRI, Muscle Force and Function Define Endpoints for Clinical Trials
Source: PLoS One. 2016 Sep 20;11(9):e0162542. doi: 10.1371/journal.pone.0162542 (PMC5029878; doi:10.1371/journal.pone.0162542)
Supplement: S1 Fig — (DOCX) [file pone.0162542.s001.docx]

**S1 Figure**

**
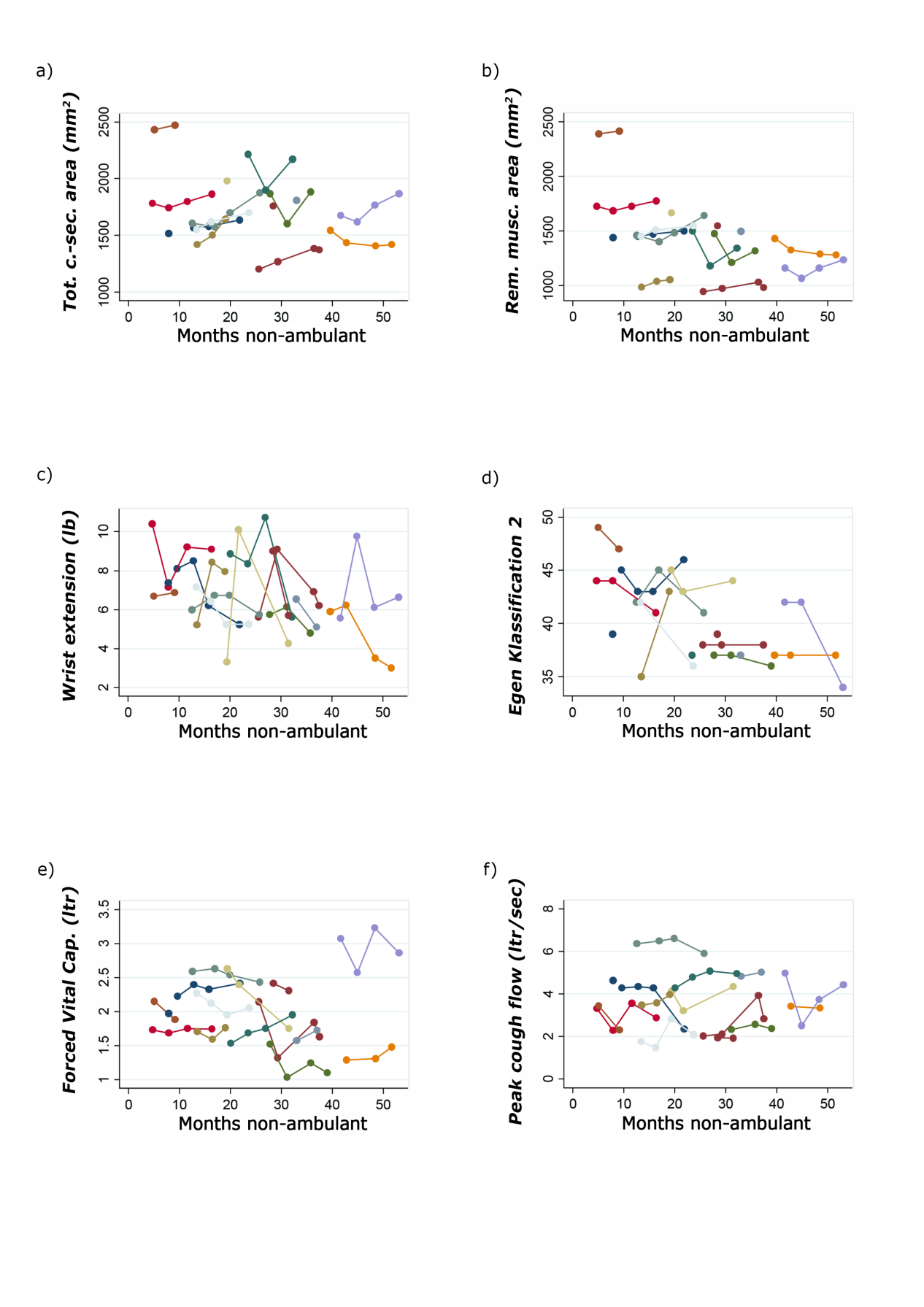
**

**S1 Figure (A) Individual trajectories for central slice total cross sectional muscle area in mm2 and (B) total remaining muscle area in mm2 , (C) wrist extension with microfet myometer (lb), (D) Egen Klassification (EK2), (E) Forced vital capacity (ltr) (F), Peak cough flow (ltr/sec)**
