# Supplementary material for: Upper Limb Evaluation in Duchenne Muscular Dystrophy: Fat-Water Quantification by MRI, Muscle Force and Function Define Endpoints for Clinical Trials
Source: PLoS One. 2016 Sep 20;11(9):e0162542. doi: 10.1371/journal.pone.0162542 (PMC5029878; doi:10.1371/journal.pone.0162542)
Supplement: S1 Table — Individual muscle-group mean fat fraction (%) for the central slice in each DMD and healthy control (HC) subject at baseline (a) and mean (95%CI) changes from baseline in DMD (b). Central slice mean f.f. at baseline and 6 weeks in healthy controls (HC) (c). (DOCX) [file pone.0162542.s002.docx]

**S1 Table**

**Table A**

| Subjects | ECU f.f. | EDM  f.f. | ED  f.f. | APL  f.f. | EPL  f.f. | ECRLB Br f.f. | FCU f.f. | FDP f.f. | FCR f.f. | FDS & PL f.f. |
| --- | --- | --- | --- | --- | --- | --- | --- | --- | --- | --- |
| 1 DMD | 3.41 | 1.14 | 3.50 | 4.58 | 3.40 | 26.41 | 5.88 | 4.99 | 4.90 | 7.09 |
| 2 DMD | 5.44 | 2.95 | 5.27 | 10.98 | 9.49 | 55.10 | E | 12.94 | 26.15 | 46.72 |
| 3 DMD | 8.56 | 12.69 | 9.86 | 19.26 | 7.56 | 13.36 | 49.10 | 16.69 | 24.19 | 34.17 |
| 4 DMD | 2.25 | 3.45 | 4.03 | 4.48 | 5.81 | 43.67 | 11.64 | 2.65 | 5.16 | 6.18 |
| 5 DMD | 1.73 | 2.44 | 4.47 | 3.21 | 4.10 | 9.76 | 22.81 | 7.74 | 34.73 | 13.39 |
| 6 DMD | 2.87 | 1.23 | 1.87 | 1.93 | 2.43 | 14.58 | 3.82 | 1.32 | 4.19 | 5.81 |
| 7 DMD | 2.21 | 7.72 | 40.36 | 25.28 | 22.94 | 54.52 | 6.97 | 35.21 | 24.01 | 47.72 |
| 8 DMD | 91.92 | 88.59 | 68.19 | 84.76 | 75.76 | 46.23 | 75.94 | 91.95 | 87.61 | 94.56 |
| 9 DMD | 0.77 | 0.00 | 0.03 | 0.29 | 0.66 | 7.84 | 3.97 | 1.58 | 0.48 | 1.52 |
| 10 DMD | 7.94 | 14.24 | 17.23 | 16.11 | 13.13 | 52.27 | 31.68 | 11.07 | 30.58 | 12.68 |
| 11 DMD | 12.69 | 11.53 | 25.37 | 23.47 | 30.19 | 30.69 | 32.30 | 22.78 | 38.58 | 47.92 |
| 12 DMD | 2.57 | 2.24 | 3.68 | 2.01 | 2.25 | 22.12 | 6.03 | 1.93 | 21.74 | 15.85 |
| 13 DMD | 2.15 | 3.18 | 4.93 | 4.96 | 5.93 | 22.34 | 6.40 | 3.66 | 6.92 | 1.99 |
| 14 DMD | 5.39 | 11.54 | 12.56 | 25.14 | 12.51 | 32.43 | 12.08 | 3.61 | 27.84 | 11.97 |
| 15 DMD | 12.96 | 13.33 | 24.29 | 35.66 | 19.79 | 37.66 | 36.40 | 27.24 | 56.73 | 41.59 |
|  | | | | | | | | | | |
| 1 HC | 0.09 | 0.47 | 1.50 | 0.72 | 0.00 | 1.67 | 2.42 | 0.50 | 1.34 | 0.93 |
| 2 HC | 1.48 | 0.63 | 0.95 | 0.28 | 1.40 | 1.03 | 1.58 | 0.59 | 0.07 | 0.24 |
| 3 HC | 0.13 | 0.52 | 1.01 | 0.53 | 1.19 | 1.20 | 0.20 | 0.11 | 0.46 | 0.58 |
| 4 HC | 1.14 | 0.68 | 2.07 | 0.64 | 0.68 | 0.78 | 2.41 | 0.58 | 1.84 | 0.88 |
| 5 HC | 4.37 | 1.39 | 4.39 | 0.06 | -1.55 | 4.70 | 0.40 | 0.16 | 0.01 | 0.01 |
| 6 HC | 2.35 | 1.18 | 2.47 | 0.19 | 0.99 | 1.31 | 2.25 | 0.73 | 2.04 | 1.82 |
| 7 HC | 0.66 | 0.80 | 0.98 | 0.48 | 0.38 | 0.25 | 3.31 | 0.44 | 0.91 | 0.93 |
| 8 HC | 1.66 | 0.60 | 1.65 | 0.14 | 3.58 | 4.30 | 0.85 | 0.86 | 0.65 | 0.61 |
| 9 HC | 1.69 | 1.31 | 1.23 | 0.98 | 2.09 | 1.50 | 1.15 | 0.37 | 2.03 | 0.68 |
| 10 HC | 1.22 | 1.04 | 1.01 | 0.70 | 0.34 | 1.34 | -0.07 | 0.57 | 0.57 | 0.38 |

**Table B**

|  | | 3 months | 6 months | 12 months |
| --- | --- | --- | --- | --- |
| ECU FAT FRACTION (%) | | | | |
|  | **Mean change from baseline (95% CI)** | 0.7  (-0.3, 1.8) | 0.7  (-0.4, 1.8) | 1.6  (0.6, 2.7) |
|  | **No. of subjects** | 9 | 7 | 8 |
|  | **P value** | 0.17 | 0.19 | <0.01 |
| EDM FAT FRACTION (%) | | | | |
|  | **Mean change from baseline (95% CI)** | 0.1  (-2.2, 2.5) | 2.2  (-0.4, 4.6) | 3.8  (1.4, 6.3) |
|  | **No. of subjects** | 9 | 7 | 8 |
|  | **P value** | 0.92 | 0.10 | <0.01 |
| ED FAT FRACTION (%) | | | | |
|  | **Mean change from baseline (95% CI)** | 1.2  (-0.7, 3.2) | 2.3  (0.2, 4.4) | 2.9  (0.9, 4.9) |
|  | **No. of subjects** | 9 | 7 | 8 |
|  | **P value** | 0.21 | 0.03 | <0.01 |
| APL FAT FRACTION (%) | | | | |
|  | **Mean change from baseline (95% CI)** | 1.2  (-2.1, 4.5) | 4.3  (0.8, 7.9) | 6.6  (3.2, 10.0) |
|  | **No. of subjects** | 9 | 7 | 8 |
|  | **P value** | 0.48 | 0.02 | <0.001 |
| EPL FAT FRACTION (%) | | | | |
|  | **Mean change from baseline (95% CI)** | 1.2  (-0.8, 3.2) | 1.0  (-1.1, 3.1) | 1.3  (-0.8, 3.3) |
|  | **No. of subjects** | 9 | 7 | 8 |
|  | **P value** | 0.23 | 0.35 | 0.22 |
| ECRLB Br FAT FRACTION (%) | | | | |
|  | **Mean change from baseline (95% CI)** | 1.7  (-1.1, 4.5) | 6.1  (3.1, 9.2) | 7.0  (4.1, 10.0) |
|  | **No. of subjects** | 9 | 7 | 8 |
|  | **P value** | 0.24 | <0.001 | <0.001 |
| FCU FAT FRACTION (%) | | | | |
|  | **Mean change from baseline (95% CI)** | 3.6  (0.6, 6.6) | 3.9  (0.6, 7.1) | 6.0  (2.9, 9.1) |
|  | **No. of subjects** | 9 | 7 | 8 |
|  | **P value** | 0.02 | 0.02 | <0.001 |
| FDP FAT FRACTION (%) | | | | |
|  | **Mean change from baseline (95% CI)** | 0.05  (-2.4, 2.5) | 1.3  (-1.3, 3.9) | 4.8  (2.3, 7.3) |
|  | **No. of subjects** | 9 | 7 | 8 |
|  | **P value** | 0.97 | 0.32 | <0.001 |
| FCR FAT FRACTION (%) | | | | |
|  | **Mean change from baseline (95% CI)** | 2.0  (-1.9, 5.9) | 2.6  (-1.6, 6.8) | 7.1  (3.1, 11.1) |
|  | **No. of subjects** | 9 | 7 | 8 |
|  | **P value** | 0.31 | 0.22 | <0.01 |
| FDS & PL FAT FRACTION (%) | | | | |
|  | **Mean change from baseline (±SD)** | 2.7  (0.3, 5.0) | 6.0  (3.5, 8.6) | 7.1  (4.7, 9.6) |
|  | **No. of subjects** | 9 | 7 | 8 |
|  | **P value** | 0.03 | <0.001 | <0.001 |

**Table C**

| Healthy control subject | Central slice f.f. (%) at baseline | Central slice f.f. (%) at 6 weeks |
| --- | --- | --- |
| 1 HC | 0.94 | 0.78 |
| 2 HC | 0.68 | 0.87 |
| 3 HC | 0.48 | 0.75 |
| 4 HC | 0.95 | 1.23 |
| 5 HC | 0.99 | - |
| 6 HC | 1.31 | - |
| 7 HC | 0.81 | - |
| 8 HC | 0.83 | - |
| 9 HC | 0.88 | - |
| 10 HC | 0.66 | - |

**S1 Table: Individual muscle-group mean fat fraction (%) for the central slice in each DMD and healthy control (HC) subject at baseline (A) and mean (95%CI) changes from baseline in DMD (B) . Central slice mean f.f. at baseline and 6 weeks in healthy controls (HC) (C)**. Extensor carpi ulnaris (ECU), extensor digiti minimi (EDM), extensor digitorum (ED), abductor pollicis longus (APL), extensor pollicis longus (EPL), extensor carpi radialis longus/brevis and brachioradialis (ECRLB Br), flexor carpi ulnaris (FCU), flexor digitorum profundus and flexor pollicis longus (FDP), flexor digitorum; flexor carpi radialis (FCR); superficialis and palmaris longus (FDS & PL). P value < 0.01 was considered significant.
