# Supplementary material for: Upper Limb Evaluation in Duchenne Muscular Dystrophy: Fat-Water Quantification by MRI, Muscle Force and Function Define Endpoints for Clinical Trials
Source: PLoS One. 2016 Sep 20;11(9):e0162542. doi: 10.1371/journal.pone.0162542 (PMC5029878; doi:10.1371/journal.pone.0162542)
Supplement: S2 Table — (DOCX) [file pone.0162542.s003.docx]

**S2 Table**

|  | | | 3 months | 6 months | 12 months |
| --- | --- | --- | --- | --- | --- |
| CENTRAL SLICE TOTAL MUSCLE COMPARTMENT FAT FRACTION (%) | | | | | |
|  | **Mean change from baseline (95% CI)** | | 1.40  (0.06, 2.73) | 3.53  (2.07, 4.98) | 3.90  (2.45, 5.35) |
|  | **No. of subjects** | | 8 | 6 | 6 |
|  | **P value** | | 0.04 | < 0.001 | < 0.001 |
| PROXIMAL SLICE TOTAL MUSCLE COMPARTMENT FAT FRACTION (%) | | | | | |
|  | **Mean change from baseline (95% CI)** | | 1.79  (0.30, 3.26) | 4.46  (2.81, 6.10) | 6.03  (4.42, 7.64) |
|  | **No. of subjects** | | 8 | 6 | 6 |
|  | **P value** | | 0.02 | < 0.001 | < 0.001 |
| DISTAL SLICE TOTAL MUSCLE COMPARTMENT FAT FRACTION (%) | | | | | |
|  | **Mean change from baseline (95% CI)** | | 1.71  (-0.30, 3.71) | 1.63  (-0.55, 3.81) | 3.20  (1.02, 5.38) |
|  | **No. of subjects** | | 8 | 6 | 6 |
|  | **P value** | | 0.10 | 0.14 | < 0.01 |
| CENTRAL SLICE DORSAL COMPARTMENT FAT FRACTION (%) | | | | | |
|  | **Mean change from baseline (95% CI)** | | 1.5  (-1.4, 4.3) | 5.0  (1.9, 8.1) | 5.4  (2.5, 8.4) |
|  | **No. of subjects** | | 8 | 6 | 7 |
|  | **P value** | | 0.32 | <0.01 | <0.001 |
| CENTRAL SLICE VOLAR COMPARTMENT FAT FRACTION (%) | | | | | |
|  | **Mean change from baseline (95% CI)** | | 1.3  (-0.6, 3.3) | 0.06  (-2.0, 2.2) | 2.1  (0.1, 4.1) |
|  | **No. of subjects** | | 8 | 6 | 7 |
|  | **P value** | | 0.18 | 0.95 | 0.04 |
| ECRLB Br FAT FRACTION (%) | | | | | |
|  | **Mean change from baseline (95% CI)** | | 1.3  (-1.6, 4.1) | 4.8  (1.8, 7.9) | 5.9  (3.0, 8.8) |
|  | **No. of subjects** | | 8 | 6 | 7 |
|  | **P value** | | 0.38 | <0.01 | <0.001 |
| CENTRAL SLICE TOTAL MUSCLE COMPARTMENT CROSS-SECTIONAL MUSCLE AREA (mm2) | | | | | |
|  | | **Mean change from baseline (95% CI)** | -17.93  (-99.68, 63.83) | 18.15  (-70.70, 106.97) | 101.47  (12.65, 190.29) |
|  |  | **No. of subjects** | 8 | 6 | 6 |
|  |  | **P value** | 0.67 | 0.69 | 0.03 |
| CENTRAL SLICE TOTAL REMAINING (NON-FAT) MUSCLE AREA (mm2) | | | | | |
|  | | **Mean change from baseline (95% CI)** | -38.65  (-110.08, 32.79) | -46.26  (-123.86, 31.34) | 15.19  (-62.41, 92.79) |
|  |  | **No. of subjects** | 8 | 6 | 6 |
|  |  | **P value** | 0.29 | 0.24 | 0.70 |
| MYOPINCH (Kg) | | | | | |
|  | | **Mean change from baseline (95% CI)** | -0.16  (-0.31, -0.004) | -0.39  (-0.55, -0.23) | -0.52  (-0.68, -0.36) |
|  |  | **No. of subjects** | 9 | 8 | 8 |
|  |  | **P value** | 0.04 | < 0.001 | < 0.001 |
| MYOGRIP (Kg) | | | | | |
|  | | **Mean change from baseline (95% CI)** | -0.20  (-0.72, 0.32) | -0.43  (-0.97, 0.12) | -1.01  (-1.56, -0.47) |
|  |  | **No. of subjects** | 9 | 8 | 8 |
|  |  | **P value** | 0.45 | 0.13 | < 0.001 |
| PERFORMANCE OF UPPER LIMB (Total score=74) | | | | | |
|  | | **Mean change from baseline (95% CI)** | -0.48  (-2.84, 1.88) | -2.60  (-5.18, -0.03) | -9.19  (-11.96, -6.42) |
|  |  | **No. of subjects** | 13 | 10 | 8 |
|  |  | **P value** | 0.69 | 0.05 | < 0.001 |
| PERFORMANCE OF UPPER LIMB ( Shoulder domain score =16) | | | | | |
|  | | **Mean change from baseline (95% CI)** | -0.37  (-2.79, 2.05) | -2.70  (-5.37, -0.03) | -8.37  (-11.26, -5.48) |
|  |  | **No. of subjects** | 13 | 10 | 8 |
|  |  | **P value** | 0.77 | 0.05 | < 0.001 |
| MOVIPLATE (taps in 30 seconds) | | | | | |
|  | | **Mean change from baseline (95% CI)** | 2.42  (-0.05, 4.90) | 5.08  (2.14, 8.02) | 1.86  (-0.82, 4.55) |
|  |  | **No. of subjects** | 10 | 8 | 8 |
|  |  | **P value** | 0.06 | 0.001 | 0.17 |

**S2 Table. MRI and clinical indices: mean (95%CI) changes from baseline; analysis of variance excluding the DMD subject not taking steroid therapy.** P value < 0.01 was considered significant.
