# Supplementary material for: Upper Limb Evaluation in Duchenne Muscular Dystrophy: Fat-Water Quantification by MRI, Muscle Force and Function Define Endpoints for Clinical Trials
Source: PLoS One. 2016 Sep 20;11(9):e0162542. doi: 10.1371/journal.pone.0162542 (PMC5029878; doi:10.1371/journal.pone.0162542)
Supplement: S3 Table — (DOCX) [file pone.0162542.s004.docx]

**S3 Table**

|  | | 3 months | 6 months | 12 months |
| --- | --- | --- | --- | --- |
| SHOULDER FLEXION (lb) | | | | |
|  | **Mean change from baseline (95% CI)** | 1.5  (-0.1, 3.0) | 0.7  (-1.0, 2.4) | -1.6  (-3.4, 0.2) |
|  | **No. of subjects** | 14 | 11 | 9 |
|  | **P value** | 0.06 | 0.41 | 0.09 |
| ELBOW FLEXION (lb) | | | | |
|  | **Mean change from baseline (95% CI)** | 0.1  (-0.7, 0.9) | -0.8  (-1.7, 0.1) | -0.8  (-1.8, 0.1) |
|  | **No. of subjects** | 14 | 11 | 9 |
|  | **P value** | 0.80 | 0.10 | 0.09 |
| ELBOW EXTENSION (lb) | | | | |
|  | **Mean change from baseline (95% CI)** | 0.8  (-0.3, 1.8) | -0.7  (-1.8, 0.4) | -1.3  (-2.4, -0.08) |
|  | **No. of subjects** | 14 | 11 | 9 |
|  | **P value** | 0.15 | 0.22 | 0.04 |
| WRIST EXTENSION (lb) | | | | |
|  | **Mean change from baseline (95% CI)** | 1.5  (-0.1, 3.0) | 0.7  (-1.0, 2.4) | -1.6  (-3.4, 0.2) |
|  | **No. of subjects** | 14 | 11 | 9 |
|  | **P value** | 0.06 | 0.41 | 0.09 |
| FORCED VITAL CAPACITY (ltr) | | | | |
|  | **Mean change from baseline (95% CI)** | -0.2  (-0.3, -0.03) | -0.1  (-0.3, 0.04) | -0.1  (-0.3, 0.05) |
|  | **No. of subjects** | 14 | 11 | 9 |
|  | **P value** | 0.02 | 0.14 | 0.18 |
| PEAK COUGHT FLOW (ltr/sec) | | | | |
|  | **Mean change from baseline (95% CI)** | -0.4  (-0.8, 0.05) | 0.3  (-0.1, 0.8) | -0.2  (-0.7, 0.3) |
|  | **No. of subjects** | 14 | 11 | 9 |
|  | **P value** | 0.09 | 0.17 | 0.37 |
| EGEN KLASSIFICATION (EK2) | | | | |
|  | **Mean change from baseline (95% CI)** | 0.2  (-1.7, 2.0) | 1.2  (-1.8, 4.2) | -1.9  (-3.9, 0.07) |
|  | **No. of subjects** | 9 | 3 | 8 |
|  | **P value** | 0.87 | 0.43 | 0.06 |
